# Clay and climatic variability explain the global potential distribution of *Juniperus phoenicea* toward restoration planning

**DOI:** 10.1038/s41598-022-16046-0

**Published:** 2022-08-01

**Authors:** Mohammed A. Dakhil, Reham F. El-Barougy, Ali El-Keblawy, Emad A. Farahat

**Affiliations:** 1 Botany and Microbiology Department, Faculty of Science, Helwan University, Cairo, 11795 Egypt; 2 Department of Botany and Microbiology, Faculty of Science, Damietta University, New Damietta, Egypt; 3 Department of Ecology and Evolutionary Biology, University of Toronto, Toronto, ON Canada; 4 Department of Applied Biology, Faculty of Science, University of Sharjah, P.O. Box 27272, Sharjah, United Arab Emirates

**Keywords:** Ecology, Biogeography, Ecological modelling

## Abstract

*Juniperus phoenicea* is a medicinal conifer tree species distributed mainly in the Mediterranean region, and it is IUCN Red Listed species, locally threatened due to arid conditions and seed over-collection for medicinal purposes, particularly in the East-Mediterranean region. Several studies have addressed the potential distribution of *J. phoenicea* using bioclimatic and topographic variables at a local or global scale, but little is known about the role of soil and human influences as potential drivers. Therefore, our objectives were to determine the most influential predictor factors and their relative importance that might be limiting the regeneration of *J. phoenicea*, in addition, identifying the most suitable areas which could be assumed as priority conservation areas. We used ensemble models for species distribution modelling. Our findings revealed that aridity, temperature seasonality, and clay content are the most important factors limiting the potential distribution of *J. phoenicea.* Potentially suitable areas of the output maps, in which *J. phoenicea* populations degraded, could be assumed as decision-support tool reforestation planning. Other suitable areas, where there was no previous tree cover are a promising tool for afforestation and conservation planning. Finally, conservation actions are needed for natural habitats, particularly in the arid and semi-arid regions, which are highly threatened by global warming.

## Introduction

Global climate change has an unprecedented impact on forests worldwide, causing changes in ecosystems functions and services, species abundance, and biodiversity^1^. Its effects include phenological changes and change local and global species distribution, increasing the risk of plant species extinction on a local and global scale^1,2^. As a result, understanding the effects of climate change on the distribution and abundance of a plant species in the current and future time is critical^3^. Understanding many environmental issues and predicting species responses to environmental change are facilitated by using the species distribution models (SDMs)^4,5^. The ability of SDMs to predict the probability of species presence has many ecological applications, such as conservation of many threatened and endangered species and prediction of suitable habitats under alternative climate change scenarios^6,7^. Ensemble modelling is an alternative to the single SDM algorithms, and through it, we use multiple algorithms simultaneously to generate an ensemble SDM^8^. The advantage of the ensemble model approach is its ability to improve the model predictions and reduce the overfitting considering the variability in individual modeling techniques' predictions^9,10^.

Soil properties significantly impact plant growth and distribution^11^. For example, several studies have reported the importance of soils in driving species distribution^12–14^. The inclusion of quantitative edaphic variables, such as pH, inorganic carbon, and volumetric soil water content, can significantly improve the prediction quality of the distribution of a single plant species^15–17^. As a result, focusing solely on climate predictors may result in an inadequate quantification of the niche of a given species. However, the availability of ecophysiologically significant variables, such as microclimate and edaphic variables, is hard to be sourced^18^. Therefore, the accurate fine-scale predictions of species distributions require developing predictors that depend on significant ecological factors, especially those better reflecting the local soil properties and climatic conditions, and allow for more^18,19^.

*Juniperus phoenicea* L. (Cupressaceae family) is an evergreen monoecious coniferous tree^20^. Its distribution covers the North African Mediterranean countries and it extends to the Arabian coast of the Red Sea toward the east and to the Canary Islands and Madeira to the west^21^. It grows on hills and dunes in North Africa and in arid mountainous regions^20^. The current status of *J. phoenicea* is recorded as Least Concern (LC) at the IUCN website (https://www.iucnredlist.org/species/16348983/99965052). The conservation status of a "least concern" species indicates neither threatened nor near-threatened within the species range. However, the species is subjected to several pressures, including overcutting for biofuel and medicinal purposes, natural habitat degradation, and repeated drought, especially in arid and semi-arid regions^14^. Although the species is found in many protected areas^22^, there is evidence of an ongoing decline of *J. phoenicea* populations in many global regions, including Egypt, where it was classified as a very rare species. During the past few decades, *J. phoenicea* has suffered from over-collection, habitat destruction, and degradation in North Sinai, Egypt^23,24^.

Furthermore, there were limited seedling recruitment and high mortality rates in both old and young individuals of *J. phoenicea* at the same sites of North Sinai. Moreover, the youngest tree was 50and 96 years old in two populations in Northern Sinai, indicating that these populations are declining^25^. The main reported important factors that limit the distribution of Juniper in North Sinai populations are altitude, topography, and soil factors, especially pH and salinity^26,27^. El-Banna^23^ and Farahat^25^ found that Juniper individuals at Sinai's Mountains have poor vitality at the high altitudes (600–1000 m a.s.l.), but better vitality, with most healthy foliage and reproductive branches at lower altitudes (350–500 m a.s.l.). Moreover, in arid and semi-arid regions (e.g., southern Saudi Arabia, Oman, and Algeria), the juniper populations are critically threatened, especially in areas facing repeated drought^14,27,28^. Accordingly, this reflects that *J. phoenicea* is locally abundant in the West Mediterranean countries while it might be highly threatened in the East Mediterranean countries and Arabian Peninsula.

The potential distribution of *J. phoenicea* had been well addressed recently using the bioclimatic variables at global scale^29^ or using bioclimatic, and soil variables at a local scale^14^. However, more attention should be paid to the effects of other relevant, meaningful variables, such as potential evapotranspiration and aridity, and human influence. Studying these factors is very important for *J. phoenicea,* which prefers growing in humid habitats, yet growing in arid regions. Therefore, conservation of this species in arid mountainous areas in the Sinai, Red Seas, and Saudia mountains is a real challenge. Another challenge facing the conservation of this species is its limited ability of dispersal and regeneration^23,25,29,30^.

Accordingly, the objectives of the present study were to (1) evaluate the relative importance of climatic, edaphic, and human-influence variables in explanation of the potential distribution of *J. phoenicea*, (2) determine the most influential predictor factors that might be limiting the regeneration of *J. phoenicea*, (3) identify the most suitable areas which could be proposed as priority conservation sites for restoration planning*.* We expect that the current study's findings will help find out the most suitable areas to support the survival of this species and be used for future conservation, especially in declining populations.

## Methods

### Species distribution data

**Figure 1:**
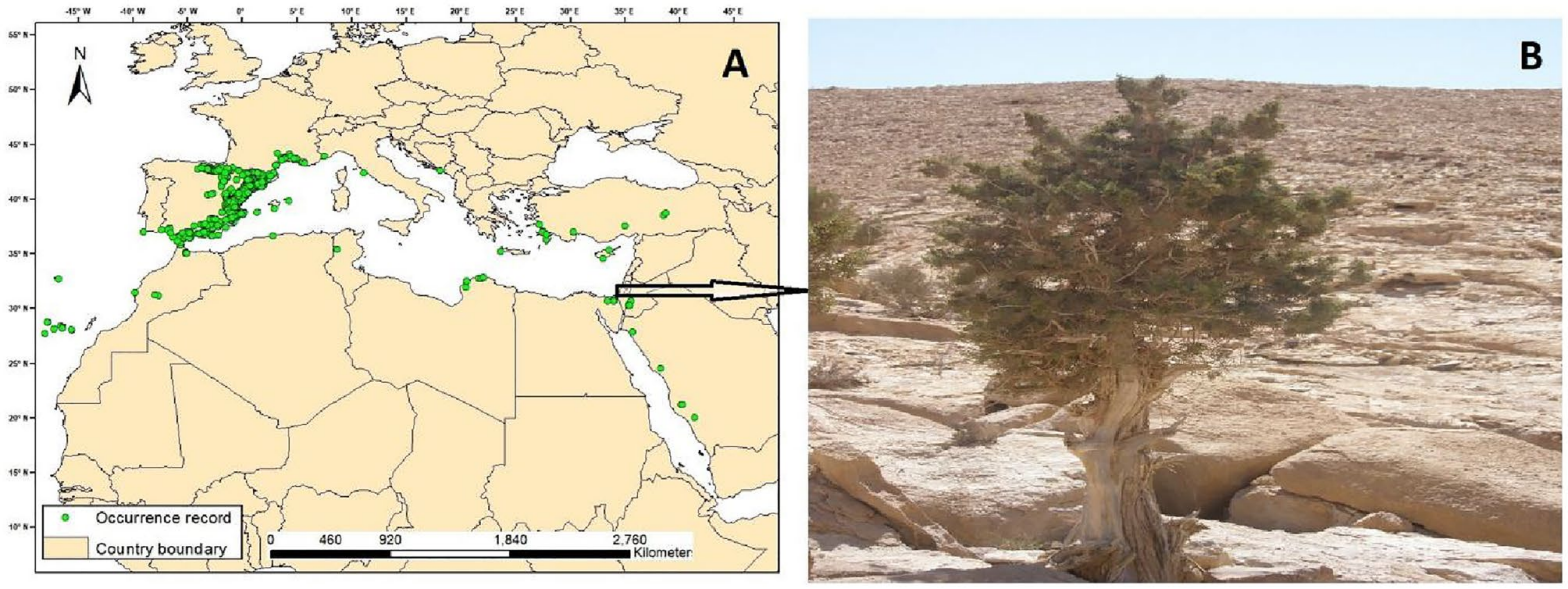
Global distribution map of *Juniperus pheonicea* generated in ArcGIS 10.5 (**A**) and a growing plant at a dry, rocky habitat in north Sinai (Egypt) (**B**).

We obtained a total of 8220 occurrence record data of *J. phoenicea* from three sources: GBIF.org (https://doi.org/10.15468/dl.abpfdb, accessed on 01 January 2021), Moustafa et al. (2016), and Farahat (2020). In ESRI ArcGIS 10.5, we removed duplicates and the records outside the shapefile of the study area (ESRI world map), resulting in 7162 occurrence records (Fig. 1A). After deleting the reciprocated missing values of the environmental variables of climate, topography, soil, and human influence, the occurrence of *J. phoenicea* was reduced further into 7067 records.

### Environmental and human influence data and multicollinearity

The digital elevation model (DEM) was obtained from the U.S. geological survey (https://www.usgs.gov) at 30 arc-seconds spatial resolution. The nineteen bioclimatic variables of the current climate (1970–2000) were taken from WorldClim v 2.0 (https://www.worldclim.org/data/) at a resolution of 30 arc-seconds. The data of potential evapotranspiration (PET), actual evapotranspiration (AET), and aridity index (AI) were downloaded from the CGIAR-CSI Global database^31^ (www.cgiarcsi.org) at a spatial resolution of 30 arc-s (~ 1 km at the equator). Then, the elevation and climate data layers were resampled into resolution 2.5 arc-min using ArcGIS 10.5. The physical and chemical soil properties were represented by nine quantitative variables downloaded from the ISRIC-World Soil Information database at 0–2 m depth and a spatial resolution of 30 arc-s^32^. We used the spatial analyst toolbox to generate the mean raster layers of the different soil depths. The layers were then resampled to 2.5 arc-min (~ 5 km) resolution using ArcGIS10.5.

The data of Global Human Modification of Terrestrial Systems (anthropogenic stressors) that could influence the terrestrial ecosystems were downloaded from the NASA Socioeconomic Data and Applications Center (SEDAC) at 1 Km^2^ spatial resolution^33^. This database comprises 13 anthropogenic stressors, including human settlement, agriculture, transportation, and infrastructures.

To avoid overfitting the models, we carried out correlation tests between all the selected 31 predictor variables (environmental and human influence variables, Supplementary Material). The uncorrelated variables were kept after using the variance inflation factor (VIF) that measures how strongly each predictor can explain the rest of the predictors^34^. The VIFcor and VIFstep functions of the package "usdm" were used to accomplish VIF analysis^35^ in R 4.1.1. This analysis helped exclude the variables with the VIF values > 5 and a correlation threshold of 0.75^36^.

The multicollinearity analysis resulted in 15 variables, including altitude, human influence, seven climatic variables, and six soil variables (Table 1). The resolution of 2.5 arc min was used to accept more flexibility of the interactive geographical relationship between the species and its environment^37^.

**Table 1:** Relative importance and range of predictor variables explaining potential distribution.

Variable	Code	Description	Relative variable importance (%)	VIF	Range	
					Min. value	Max. value
Soil	aw	Available soil water capacity	0.1	2.14	11.00	17.0
	cec	Cation exchange capacity in cmolc/kg	3.4	3.83	11.00	42.00
	**clay**	Soil texture fraction clay in percent	**14**	2.77	13.00	44.00
	coarse	Coarse fragments volumetric in percent	3.6	1.96	2.00	48.00
	pH	Soil pH × 10 in H_2_O	2.2	3.19	56.00	81.00
	silt	Soil texture fraction silt in percent	6.9	1.40	20.00	45.00
Climate	**ai**	Aridity index	**9.2**	4.63	0.00	0.25
	pet	Potential evapotranspiration (mm)	**34.8**	3.42	541	1764
	**bio3**	Isothermality (°C) (*100)	**16.5**	3.06	27.46	79.24
	**bio4**	Temperature seasonality (standard deviation *100)	**8.6**	3.87	49.25	990.77
	bio8	Mean temperatures of the wettest quarter * (°C)	1.4	4.50	1.31	27.59
	bio9	Mean temperature of the driest quarter (°C)	0.9	2.28	1.26	28.41
	**bio18**	Precipitation of the warmest quarter (mm)	**15.2**	3.17	0.00	262
Human influences	human	Human modification system (influence index)	1.8	1.52	0.07	0.91
Topography	alt	Altitude (m)	0.1	2.86	3.00	2385
**Ensemble-model threshold and accuracy**						
AUC	0.98					
TSS	0.95					
MTSS threshold	0.47					

The most important variables and their values are shown in bold.

VIF, variance inflation factor; TSS, true skill statistic; AUC, area under the curve indicate the accuracy of the ensemble models; MTSS, maximum training sensitivity plus specificity threshold.

*The term quarter means the mean temperatures during the wettest three months of the year.

### Ensemble modeling and model accuracy

The ensemble-modeling (EM) approach was combined with the Generalized Linear Model (GLM), Random Forest (RF), and Boosted Regression Trees (BRT), which have high stability and transferability^5,38^. We projected each model under the current climate-related data using 70% and 30% of the training data and performance evaluation, respectively. The availability of data on both species' presence and available environment-related data (pseudo-absence data) are essential to achieve the most effective SDMs. Thus, the number of pseudo absences was randomly sampled for each species and equaled ten times the number of presences^39,40^.

The ensemble modeling (EM) approach reduces uncertainty in the model predictions. The EM is prevalent to standard models in optimizing the model performance and transferability^41,42^. We weighted the ensemble models by the True Skill Statistic (TSS) using the "*sdm*" package in R 4.1.1^34^. The maximum training sensitivity plus specificity (MTSS) was used as a recommended threshold to minimize commission and omission errors^43,44^. The value of the area under the curve (AUC) closer to 1 demonstrates superior model performance^45^. The evaluation of the model performance was also assessed by True Skill Statistic (TSS) that assesses the model accuracy^45,46^. The TSS is better than AUC as it is threshold-dependent and accounts for both sensitivity and specificity, with values ranging from 0 to 1^45^.

## Results

### Model performance

The ensemble models showed excellent fits and high performance in predicting *J. phoenicea* distribution. Although we have used many environmental predictor variables of climate, soil, and human influence, the model performance fitted perfectly with mean values of AUC and TSS greater than 0.95 (Table 1), indicating the high preference of *J. phoenicea* to the climate and soil conditions (environmental filters).

### Global potential suitability of J. phoenicea

**Figure 2:**
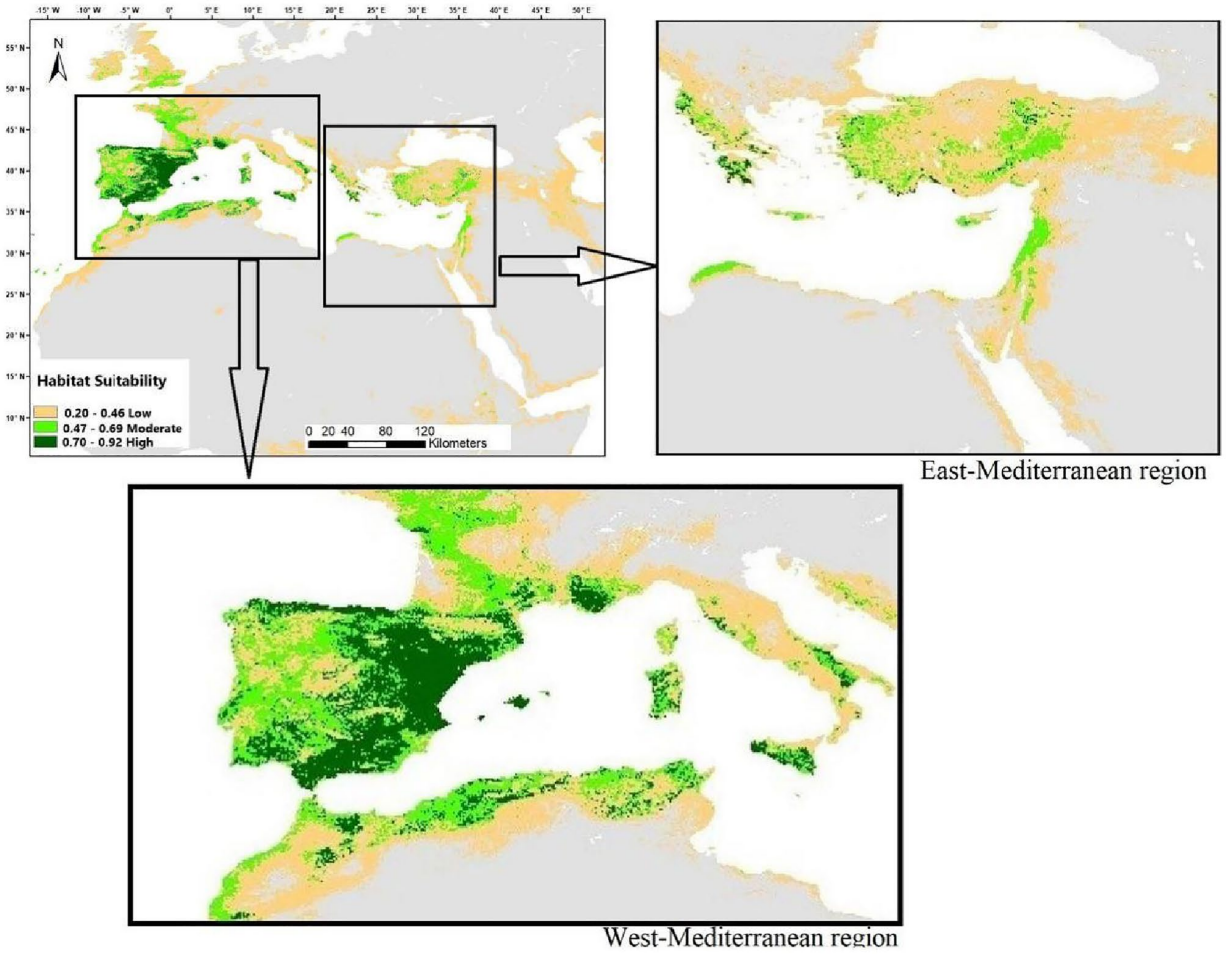
Global potential habitat suitability generated from the ensemble modelling and visualized in ArcGIS 10.5 using the maximum training sensitivity plus specificity threshold (MTSS).

Most of the presence records were found in the West-Mediterranean region, particularly in Spain (Fig. 1), with the highest potential suitable areas compared to other countries. Moreover, some countries of the West-Mediterranean region, either with very few occurrence records or without any records, showed high suitability, such as Tunisia, Algeria, Morocco, France, Portugal, Italy, and Malta. On the other hand, the East-Mediterranean region showed relatively less potential suitability, which may be attributed to the few occurrence records. Still, some countries of the East-Mediterranean region, such as Turkey, Greece, Lebanon, Palestine, and Jordan, with very few occurrence records, showed high suitability compared to other countries, particularly of the South-Mediterranean region, such as Egypt and Libya (Fig. 2).

### Key factors determining the potential global distribution of J. phoenicea

Based on the relative importance of the predictor variables generated by the ensemble models, potential evapotranspiration (PET) was the most important climatic factor contributed with 34.8% of the potential distribution of *J. phoenicea* (Table 1), followed by isothermality (Bio3, 16.5%), and precipitation of the warmest quarter (Bio18, 15.2%), aridity index (AI, 9.2%), and finally temperature seasonality (Bio4, 8.6%). Also, the percent of soil texture clay fraction was a critical predictor variable contributed by 14% in the probability of the species presence (Table 1).

**Figure 3:**
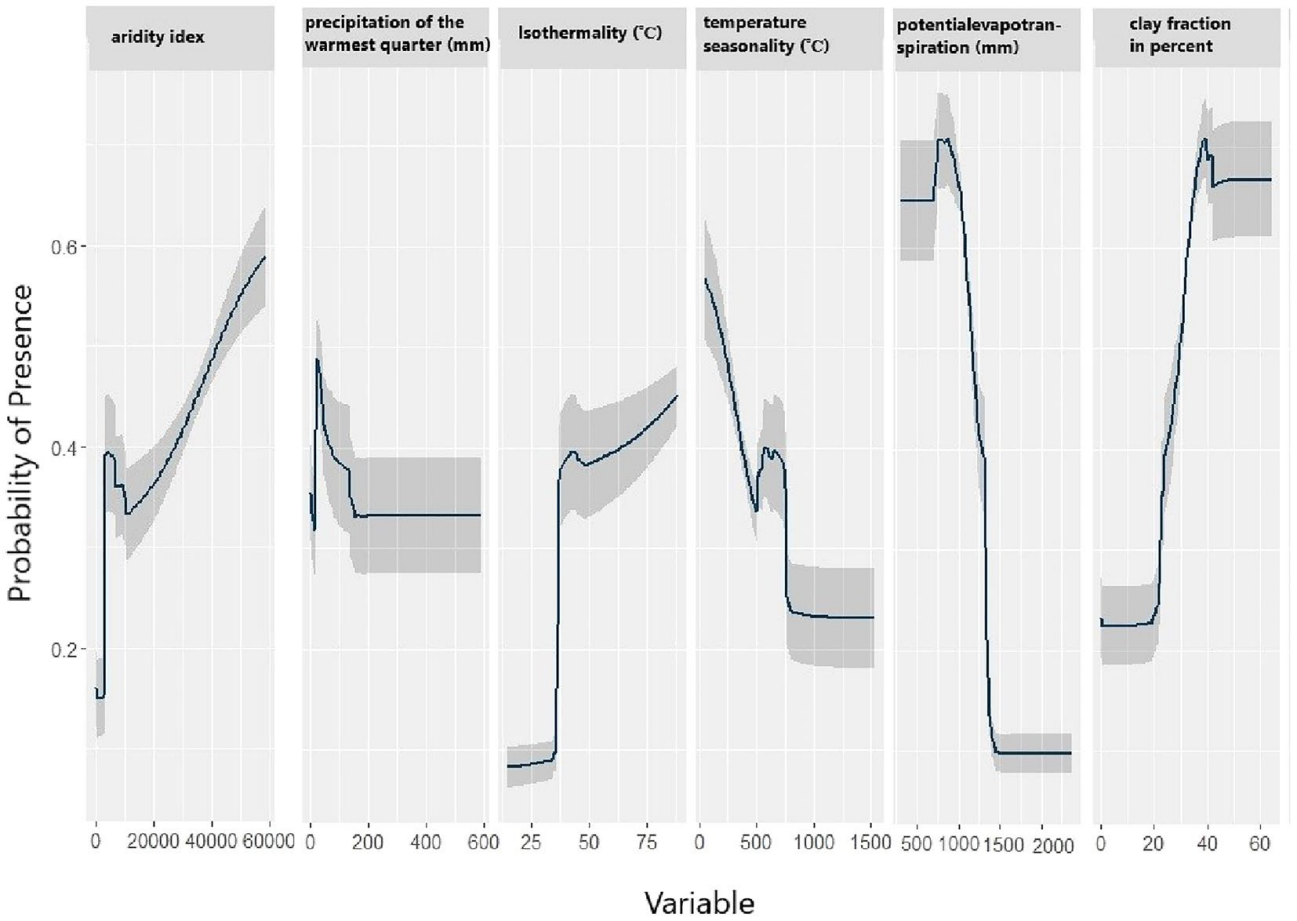
Response curves of the most important predictor variables explaining the potential global distribution of *Juniperus phoenicea*.

The response curves showed that the maximum presence probability of *J. phoenicea* was at the narrow range of PET, from 500 mm to 900 mm. A sharp decline in the presence probability was up to 1500 mm, after which it became constant (Fig. 3). Furthermore, the likelihood of *J. phoenicea* occurrence decreased gradually with the increase of temperature seasonality (Bio4), but the presence probability increased at a narrow range of approximately 5–7.5 °C. On the other hand, the potential occurrence suitability of *J. phoenicea* showed a gradual increase with the increase of aridity index ranging from 0.1 up to 0.6 (i.e., towards humid climate), indicating the preferability of *J. phoenicea* to humid habitats (Fig. 3). Moreover, the potential presence of the species increases with the increase of clay content, particularly at the narrow range (30–40%) (Fig. 3).

## Discussion

As evidenced by an independent test dataset, the selected environmental variables in this study resulted in a well-performed model with high AUC and TSS, indicating a high predictive accuracy^46^ for the potential distribution of *J. phoenicea*. This study revealed that predictor variables with narrow ranges such as clay content, temperature seasonality, and potential evapotranspiration could be considered limiting factors for *J. phoenicea* potential distribution. The total contribution of PET and AI was 44%. In addition, the temperature-related variables (Bio3 and Bio4) contributed with 25.1%, soil clay fraction with 14%, and precipitation of the warmest quarter (Bio18) with 15.2% of the variation in the potential distribution *J. phoenicea*. On the other hand, very low contribution percentages represented human influence and topography. Such results indicate the negative impact of high temperature and low precipitation on the distribution of *J. phoenicea* in the world. Significantly, changes in PET, Bio4, and aridity are associated with global warming, inferring a significant impact of climate change on the potential distribution of *J. phoenicea*. Similarly^29^, found that temperature-defined climatic factors (Bio1–Bio11) explain approximately 24% of the current global potential distribution of *J. phoenicea* L. *sensu stricto (*s.s*.),* while precipitation factors (Bio12–Bio19) explain more than 75% of the distribution. Bio 19 had a high contribution (precipitation during the coldest quarter, 30.9%), and it was the main driving factor for the ecological niche of the species.

At the local scale, the main key factors for the distribution of *J. phoenicea* in Algeria were the total soil carbon (22.1%), driest month precipitation (Bio14, 19.2%), slope (11.1%), seasonality of precipitation (Bio15, coefficient of variation) (10.3%), total soil nitrogen (7%), and soil available water capacity during summer (6.3%)^14^. Besides, it was reported that the populations of this species undergo severe drought conditions and showed dieback of its foliage in many countries such as Egypt^24,25,30^, Libya^47^, Algeria^14^, as well as few sites in Spain^48,49^. Cramer^50^ reported that the future global warming in the Mediterranean region is expected to exceed global rates by 25%, indicating that the conservation priority should be given for the arid and semi-arid North-Mediterranean African and Middle East countries, in addition to Southern European countries; the temperature increase has been projected to range between 2 °C and 4 °C by the 2080s in Southern Europe^51^. This provides an urgent need for conservation planning such as reforestation or afforestation programs for this endangered species in its natural habitats or the potential climatically and edaphically suitable habitats based on the ensemble model output of the current study.

Based on this literature, extreme drought episodes (high temperature, very low precipitation) are the main reason behind the current deterioration of the *J. phoenicea* populations. Accordingly, the impact of drought seems to be a more pronounced factor at the southern distribution range of the species where high temperature, low precipitation, and severe drought episodes are more frequent than the western Mediterranean sites^52,53^. The presence of more potential suitable habitats for *J. phoenicea* by increasing the value of the aridity index, i.e., more humid climate and more soil clay content, could explain its extensive distribution in the West-Mediterranean region compared to the East-Mediterranean region. The sensitive response of *J. phoenicea* to aridity determines its probability of occurrence by previous studies such as^54^. Besides, the presence of high soil clay content help in more retention of water and support the growth of the plants for a long time compared to sandy or rocky soil that is predominant in many areas of *J. phoenicea* in the eastern Mediterranean countries^25,47^. It is apparent from the results that altitude is not a limiting factor for the distribution of *J. phoenicea* and human influences. On the contrary, we believe, according to our observations in many field sites, that the human influences, including destruction, grazing, wood, and seed collections, have a strong effect on the distribution and regeneration of the species^25,30,47^, but it masked by the high contribution percentages of other variables in the analysis.

In conclusion, it is revealed from the results that the area of the potential current distribution of *J. phoenicea* in the West Mediterranean region is still higher than that in the East Mediterranean. This entails instant conservation and protection of the current distribution areas of declined *J. phoenicea* populations in eastern Mediterranean countries, including Egypt, Jordon, Saudi Arabia, Libya, and Algeria. Restoration actions, including reforestation and afforestation, should be applied particularly in the arid and semi-arid ecosystems . Strict regulations must be put in place to prevent the logging of juniper's wood and the collection of its seeds for medicinal and commercial purposes. Moreover, due to the greater sensitivity of this species to hotter droughts^49^, its responses to the predicted future global warming should be investigated at a global scale under different scenarios of climate change.

## Supplementary Information

Supplementary Information.

## Data Availability

All data generated or analysed during this study are included in this published article and its supplementary information files.

## References

[1] Pecl GT (2017). Biodiversity redistribution under climate change: Impacts on ecosystems and human well-being. Science (80-).

[2] Walther GR (2002). Ecological responses to recent climate change. Nature.

[3] Thuiller W (2011). Consequences of climate change on the tree of life in Europe. Nature.

[4] Zimmermann NE, Edwards TC, Graham CH, Pearman PB, Svenning J (2010). New trends in species distribution modelling. Ecography (Cop.).

[5] Norberg A (2019). A comprehensive evaluation of predictive performance of 33 species distribution models at species and community levels. Ecol. Monogr..

[6] Smeraldo S (2021). Generalists yet different: Distributional responses to climate change may vary in opportunistic bat species sharing similar ecological traits. Mamm. Rev..

[7] Sohlström EH (2022). Future climate and land-use intensification modify arthropod community structure. Agric. Ecosyst. Environ..

[8] Araújo MB, New M (2007). Ensemble forecasting of species distributions. Trends Ecol. Evol..

[9] Stohlgren TJ (2010). Ensemble habitat mapping of invasive plant species. Risk Anal. Int. J..

[10] Meller L (2014). Ensemble distribution models in conservation prioritization: from consensus predictions to consensus reserve networks. Divers. Distrib..

[11] Dubuis A (2013). Improving the prediction of plant species distribution and community composition by adding edaphic to topo-climatic variables. J. Veg. Sci..

[12] Walthert L, Meier ES (2017). Tree species distribution in temperate forests is more influenced by soil than by climate. Ecol. Evol..

[13] Figueiredo FOG (2018). Beyond climate control on species range: The importance of soil data to predict distribution of Amazonian plant species. J. Biogeogr..

[14] Arar A, Nouidjem Y, Bounar R, Tabet S, Kouba Y (2020). Potential future changes of the geographic range size of Juniperus phoenicea in Algeria based on present and future climate change projections. Contemp. Probl. Ecol..

[15] Coudun C, Gégout J, Piedallu C, Rameau J (2006). Soil nutritional factors improve models of plant species distribution: An illustration with Acer campestre (L.) in France. J. Biogeogr..

[16] Buri A (2020). What are the most crucial soil variables for predicting the distribution of mountain plant species? A comprehensive study in the Swiss Alps. J. Biogeogr..

[17] Buri A (2017). Soil factors improve predictions of plant species distribution in a mountain environment. Prog. Phys. Geogr..

[18] Mod HK, Scherrer D, Luoto M, Guisan A (2016). What we use is not what we know: environmental predictors in plant distribution models. J. Veg. Sci..

[19] Scherrer D, Guisan A (2019). Ecological indicator values reveal missing predictors of species distributions. Sci. Rep..

[20] Boulos, L. *Flora of Egypt, Vol. 1*. vol. 1 (Al Hadara Publishing, 1999).

[21] Farjon, A. & Filer, D. *An atlas of the world’s conifers: An analysis of their distribution, biogeography, diversity and conservation status*. (Brill, 2013).

[22] Allen, DJ. *Juniperus phoenicea*. The IUCN red list of threatened species 2017: e.T16348983A99965052. 10.2305/IUCN.UK.2017-2.RLTS. T16348983A99965052.en. Downloaded on 19 May 2020

[23] El-Bana M, Shaltout K, Khalafallah A, Mosallam H (2010). Ecological status of the Mediterranean Juniperus phoenicea L. relicts in the desert mountains of North Sinai Egypt. Flora-Morphol. Distrib. Funct. Ecol. Plants.

[24] Moustafa A (2016). Ecological Prominence of *Juniperus phoenicea* L. Growing in Gebel Halal, North Sinai Egypt. Catrina Int. J. Environ. Sci..

[25] Farahat EA (2020). Age structure and static life tables of the endangered Juniperus phoenicea L. in North Sinai Mountains, Egypt. J. Mt. Sci..

[26] El-Wahab A (2008). Condition assessment of plant diversity of Gebel Maghara, North Sinai, Egypt. Catrina Int. J. Environ. Sci..

[27] Youssef AM, Morsy AA, Mosallam HA, Hashim AM (2014). Vegetation and soil relationships in some wadis from the North-Central part of Sinai Peninsula Egypt. Minia Sci. Bull..

[28] Fisher M (1997). Decline in the juniper woodlands of Raydah Reserve in southwestern Saudi Arabia: A response to climate changes?. Glob. Ecol. Biogeogr. Lett..

[29] Salvà-Catarineu M (2021). Past, present, and future geographic range of the relict Mediterranean and Macaronesian *Juniperus phoenicea* complex. Ecol. Evol..

[30] Quevedo L, Rodrigo A, Espelta JM (2007). Post-fire resprouting ability of 15 non-dominant shrub and tree species in Mediterranean areas of NE Spain. Ann. For. Sci..

[31] Trabucco A, Zomer RJ (2009). Global aridity index (global-aridity) and global potential evapo-transpiration (global-PET) geospatial database. CGIAR Consort. Spat. Inf..

[32] Hengl T (2014). SoilGrids1km—Global soil information based on automated mapping. PLoS One.

[33] Kennedy, C. M., Oakleaf, J. R., Theobald, D. M., Baruch-Mordo, S. & Kiesecker, J. Documentation for the global human modification of terrestrial systems (2020).

[34] Naimi B, Araújo MB (2016). sdm: a reproducible and extensible R platform for species distribution modelling. Ecography (Cop.).

[35] Naimi B (2015). usdm: Uncertainty analysis for species distribution models. R Packag. Version.

[36] Guisan, A., Thuiller, W. & Zimmermann, N. E. In *Habitat Suitability and Distribution Models: With Applications in R*. (Cambridge University Press, 2017).

[37] Dakhil MA (2021). Global invasion risk assessment of *Prosopis juliflora* at biome level : Does soil matter?. Biology.

[38] Iturbide M, Bedia J, Gutiérrez JM (2018). Background sampling and transferability of species distribution model ensembles under climate change. Glob. Planet. Change.

[39] Barbet-Massin M, Jiguet F, Albert CH, Thuiller W (2012). Selecting pseudo-absences for species distribution models: How, where and how many?. Methods Ecol. Evol..

[40] Zhang Z, Mammola S, Xian W, Zhang H (2020). Modelling the potential impacts of climate change on the distribution of ichthyoplankton in the Yangtze Estuary, China. Divers. Distrib..

[41] Thuiller W, Guéguen M, Renaud J, Karger DN, Zimmermann NE (2019). Uncertainty in ensembles of global biodiversity scenarios. Nat. Commun..

[42] Breiner FT, Nobis MP, Bergamini A, Guisan A (2018). Optimizing ensembles of small models for predicting the distribution of species with few occurrences. Methods Ecol. Evol..

[43] Liu C, Newell G, White M (2016). On the selection of thresholds for predicting species occurrence with presence-only data. Ecol. Evol..

[44] Haider SM, Benscoter AM, Pearlstine L, D’Acunto LE, Romañach SS (2021). Landscape-scale drivers of endangered Cape Sable Seaside Sparrow (Ammospiza maritima mirabilis) presence using an ensemble modeling approach. Ecol. Modell..

[45] Allouche O, Tsoar A, Kadmon R (2006). Assessing the accuracy of species distribution models: Prevalence, kappa and the true skill statistic (TSS). J. Appl. Ecol..

[46] Franklin J (2010). Mapping Species Distributions: Spatial Inference and Prediction.

[47] Kabiel HF, Hegazy AK, Lovett-Doust L, Al-Rowaily SL, Al Borki AENS (2016). Ecological assessment of populations of *Juniperus phoenicea* L. in the Al-Akhdar mountainous landscape of Libya. Arid L. Res. Manag..

[48] Camarero JJ (2020). Dieback and mortality of junipers caused by drought: Dissimilar growth and wood isotope patterns preceding shrub death. Agric. For. Meteorol..

[49] Sánchez-Salguero R, Camarero JJ (2020). Greater sensitivity to hotter droughts underlies juniper dieback and mortality in Mediterranean shrublands. Sci. Total Environ..

[50] Cramer W (2018). Climate change and interconnected risks to sustainable development in the Mediterranean. Nat. Clim. Chang..

[51] Forzieri G (2014). Ensemble projections of future streamflow droughts in Europe. Hydrol. Earth Syst. Sci..

[52] González-Hidalgo JC (2018). High-resolution spatio-temporal analyses of drought episodes in the western Mediterranean basin (Spanish mainland, Iberian Peninsula). Acta Geophys..

[53] Stockhecke M (2016). Millennial to orbital-scale variations of drought intensity in the Eastern Mediterranean. Quat. Sci. Rev..

[54] Navarro Cerrillo RM (2021). Can habitat prediction models contribute to the restoration and conservation of the threatened tree *Abies pinsapo* Boiss. in Southern Spain?. New For..

